# Health records ‘big data’ in mental health research – should ‘Clinical Informatics’ be considered a discipline within Epidemiology?

**DOI:** 10.1192/j.eurpsy.2025.147

**Published:** 2025-08-26

**Authors:** R. Stewart

**Affiliations:** King’s College London, London, United Kingdom

## Abstract

**Background:**

Traditionally, the discipline of Epidemiology, regardless of clinical specialty, has tended to focus on public health in community populations, although its study designs are widely applied in more clinically oriented research. The emergence and rapid accumulation of digital health records have resulted in data resources that are both large in sample size and granular in detail presenting unprecedented opportunities for understanding course and outcome in mental health.

**Methods:**

Drawing on over 15 years of experience in building and achieving research output from electronic mental health records data in south London, this presentation will consider the role of Epidemiology as a discipline in this field.

**Results:**

There is no fundamental difference between studying disease incidence in community populations and using healthcare data to study disease course and outcome in clinical populations, and there are similar considerations of sample representativeness and cohort cohesion. Psychometrics principles are also strongly applicable to measurement issues in clinical data, although computer science collaborations particularly underpin the natural language processing and m-health advances required to improve information availability in routine data. Clinical Informatics and Epidemiology face common analytic challenges from data density and complexity, as well as in realising novel clinical trials opportunities.

**Conclusions:**

Clinical Informatics does benefit from the public health focus that Epidemiology brings, as well as its methodological frameworks. However, multiple disciplines are key to setting up and maintaining data resources and achieving research output, so it is equally important to flatten hierarchies and enable a genuine cross-cutting team science approach.

**Disclosure of Interest:**

R. Stewart Grant / Research support from: GSK, Takeda

